# Ferumoxytol-enhanced cardiovascular magnetic resonance detection of early stage acute myocarditis

**DOI:** 10.1186/s12968-019-0587-7

**Published:** 2019-12-16

**Authors:** Yuko Tada, Atsushi Tachibana, Shahriar Heidary, Phillip C. Yang, Michael V. McConnell, Rajesh Dash

**Affiliations:** Department of Medicine (Cardiovascular Medicine), Stanford University School of Medicine, 300 Pasteur Drive, Stanford, CA 94305 USA

**Keywords:** Ferumoxytol, CMR, MRI, Myocarditis, T2* map

## Abstract

**Background:**

The diagnostic utility of cardiovascular magnetic resonance (CMR) is limited during the early stages of myocarditis. This study examined whether ferumoxytol-enhanced CMR (FE-CMR) could detect an earlier stage of acute myocarditis compared to gadolinium-enhanced CMR.

**Methods:**

Lewis rats were induced to develop autoimmune myocarditis. CMR (3 T, GE Signa) was performed at the early- (day 14, *n* = 7) and the peak-phase (day 21, *n* = 8) of myocardial inflammation. FE-CMR was evaluated as % myocardial dephasing signal loss on gradient echo images at 6 and 24 h (6 h- & 24 h-FE-CMR) following the administration of ferumoxytol (300μmolFe/kg). Pre- and post-contrast T2* mapping was also performed. Early (EGE) and late (LGE) gadolinium enhancement was obtained after the administration of gadolinium-DTPA (0.5 mmol/kg) on day 14 and 21. Healthy rats were used as control (*n* = 6).

**Results:**

Left ventricular ejection fraction (LVEF) was preserved at day 14 with inflammatory cells but no fibrosis seen on histology. EGE and LGE at day 14 both showed limited myocardial enhancement (EGE: 11.7 ± 15.5%; LGE: 8.7 ± 8.7%; both *p* = ns vs. controls). In contrast, 6 h-FE-CMR detected extensive myocardial signal loss (33.2 ± 15.0%, *p* = 0.02 vs. EGE and *p* < 0.01 vs. LGE). At day 21, LVEF became significantly decreased (47.4 ± 16.4% vs control: 66.2 ± 6.1%, *p* < 0.01) with now extensive myocardial involvement detected on EGE, LGE, and 6 h-FE-CMR (41.6 ± 18.2% of LV). T2* mapping also detected myocardial uptake of ferumoxytol both at day 14 (6 h R2* = 299 ± 112 s^− 1^vs control: 125 ± 26 s^− 1^, *p* < 0.01) and day 21 (564 ± 562 s^− 1^, *p* < 0.01 vs control). Notably, the myocardium at peak-phase myocarditis also showed significantly higher pre-contrast T2* (27 ± 5 ms vs control: 16 ± 1 ms, *p* < 0.001), and the extent of myocardial necrosis had a strong positive correlation with T2* (*r* = 0.86, *p* < 0.001).

**Conclusions:**

FE-CMR acquired at 6 h enhance detection of early stages of myocarditis before development of necrosis or fibrosis, which could potentially enable appropriate therapeutic intervention.

## Subject terms

Inflammatory heart disease, Cardiovascular Magnetic Resonance Imaging (CMR)

## Background

Myocarditis is reported to account for up to 10–20% of acute-onset heart failure, sudden death among young adults and athletes, and unexplained cardiomyopathies [1–5]. However, the precise incidence and prevalence of myocarditis remain poorly characterized because of challenges in diagnostic accuracy [6, 7]. Current guidelines recommend the use of cardiovascular magnetic resonance (CMR) and myocardial biopsy for the diagnosis of myocarditis in patients with heart failure [8]. However, the usability of biopsy is limited because of its invasiveness and low sensitivity, with only 40% sensitivity for myocarditis [9–11]. Instead, non-invasive imaging approaches are used - with varying success - to diagnose inflammatory cardiac diseases, quantify disease activity, guide therapeutic interventions, and predict disease progression.

CMR characterizes many of the histological changes caused by myocarditis. Edematous lesions, hyperemia/vascular leakage, and necrotic or scarred tissue can be detected using T2-weighted (T2w) imaging, early gadolinium enhancement (EGE), and late gadolinium enhancement (LGE) imaging, respectively [12, 13]. The accuracy of combining T2w imaging and LGE in diagnosing myocarditis is reported to be 78% [14]. However, the specificity of CMR for inflammation is still controversial and the sensitivity for detecting reversible myocardial injury in the early inflammatory phase is relatively low [15]. Global myocardial pathologies such as diffuse inflammation or fibrosis are also difficult to delineate when using CMR techniques that depend on comparison to unaffected myocardium. Although T2w imaging is more specific for active inflammation, interpretation of T2w imaging tends to be qualitative and remains challenging.

Superparamagnetic iron oxide (SPIO) particles have been used to label myocardial cellular infiltrates to be detected on CMR [16]. SPIO administered intravenously is delivered to the interstitium by non-specific vesicular transport and through trans-endothelial channels, and taken up by inflammatory cells. Because of the superparamagnetic effects of the agents, labeled cells are detected as signal loss on T2*-weighted sequences. A longer half-life in the blood (15 h) and wider distribution of ultra-small SPIO (USPIO) are advantageous in visualizing inflammatory cellular infiltrates [17]. Ferumoxytol, an FDA-approved USPIO, has been already used clinically as an CMR contrast agent, with rates of adverse event and anaphylaxis reported to be as low as 0.2 and 0.02%, respectively [18]. The enhancement obtained from 6 to 24 h has been utilized to visualize inflammatory cells in atherosclerosis or myocardial infarction, which correlates with disease activity [19–21]. However, it remains unknown whether ferumoxytol-enhanced CMR (FE-CMR) has higher sensitivity and specificity for the diagnosis of early and active phases of myocarditis compared to gadolinium-enhanced CMR. In the preclinical rodent myocarditis model, detectability of inflammation at specific timepoints can be tested, compared between contrast agents, and verified by histological evaluation accurately. This study adopted the well-established rat autoimmune myocarditis model, which reaches an inflammatory peak at approximately day 21 post immunization and contains similar types of infiltrating cells compared to human myocarditis [22, 23]. We divided the rats into three groups; control without myocarditis, early stage day 14, and advanced stage day 21, and we performed FE-CMR, T2* mapping and gadolinium-enhanced CMR at each stage of myocarditis.

## Methods

### Rat experimental autoimmune myocarditis model

The animal protocols in this research were approved by the Stanford University Administrative Panel on Laboratory Animal Care. Male Lewis rats (*n* = 21, 6 weeks of age, 101-125 g) were obtained from Charles River Laboratories (Wilmington, Massachusetts, USA). Rats were fed a standard diet and water and maintained in compliance with animal welfare guidelines of the Institute of Experimental Animals. Experimental autoimmune myocarditis was induced by immunizing rats with purified porcine cardiac myosin (Sigma Aldrich, St. Louis, Missouri, USA). The cardiac myosin was emulsified with complete Freund’s adjuvant (DIFCO, Sparks, Maryland, USA) supplemented with *Mycobacterium tuberculosis* (DIFCO) at a concentration of 10 mg/ml. 0.2 ml of emulsion (immunizing dose of 1.0 mg of cardiac myosin per rat) was injected into the rat footpads subcutaneously [24]. During the procedure, rats were anesthetized by inhaled isoflurane (1.0–3.0%). For analgesia, buprenorphine (0.05 mg/kg) was injected subcutaneously before and after the procedure as needed.

### CMR

CMR was performed at 3 T (Signa EXCITE, General Electric Healthcare, Waukesha, Wisconsin, USA) scanner and a phased array 2 channel surface coil (Rapid MR international, Columbus, Ohio, USA). Rats were anesthetized during the scan using 1.0–3.0% of isoflurane. CMR was done under the optimized electrocardiogram (ECG) gating monitored and controlled by PC-SAM (SA Instrument, Stoneybrook, New York, USA). Heart rates during the scan were between 300 and 400/min.

**Fig. 1:**
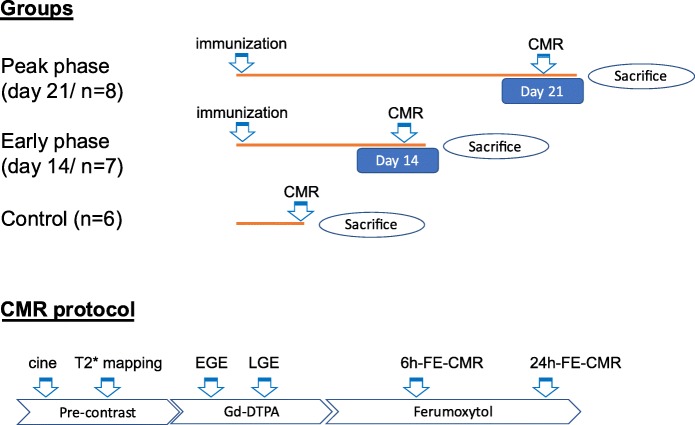
Study design The schema shows the timeline for each group and the CMR protocol

The rats were evaluated at early- (day 14) (*n* = 7) and peak-phase (day 21) (*n* = 8) of myocardial inflammation. The study design is shown in Fig. 1. Cardiac function was evaluated by cine-CMR. For cine CMR, FSPGR sequence (flip angle = 45°, TE = 10 ms, TR = 20 ms, NEX = 4, matrix = 256 × 192, FOV = 6-7 cm, thickness = 1.5 mm, BW = 122 Hz/pixel, phases to reconstruct = 20) was used to obtain 5–6 slices covering through the base to apex. Multi-slice gradient echo CMR (GRE) images were taken on the left ventricular (LV) short axis planes for T2* mapping. GRE images at different echo times (TE) times (4.9, 6.8, 9.0, 11.3, 13.5, 15.8, 18.0 ms) were used to acquire multiple short-axis images of the mid LV (flip angle =35°, TR = 1RR, trigger delay = 12 ms, NEX = 6, matrix = 256 × 192, FOV = 4-5 cm, thickness = 1.5 mm, BW = 122 Hz/pixel). For gadolinium-enhanced CMR, 0.5 mmol/kg of Gd-DTPA (Magnevist, Bayer Health Care Pharma AG, Berlin, Germany) was injected through the tail vein, and EGE and LGE were obtained. EGE was obtained with ECG-triggered T1-weighted sequence at the mid LV immediately after the gadolinium injection (TE = 16 ms, TR = 1RR, NEX = 5, matrix = 256 × 192, FOV = 4-5 cm, thickness = 1.5 mm, BW = 61 Hz/pixel). LGE was evaluated on ECG-triggered IR-FSPGR sequence (flip angle =30°, TE = 4.4 ms, TR = 12.9 ms, NEX = 5, matrix = 256 × 192, FOV = 4-5 cm, thickness = 1.5 mm, BW = 61 Hz/pixel, TI = 180-200 ms) performed during 10–20 min after the injection of Gd-DTPA. After gadolinium-enhanced CMR, ferumoxytol (Feraheme; AMAG Pharmaceuticals, Inc. Lexington, Massachusetts, USA) was administered into the rat tail vein or the femoral vein. Ferumoxytol (300μmolFe/kg) was administered based on the manufacture’s instruction and the previous reports [25, 26]. Dephasing signal loss was detected on GRE twice at 6 and 24 h later for each rat after washout of Gd-DTPA was verified. The same studies were performed in control rats (*n* = 6) that were not induced with myocarditis.

#### CMR analysis

Cine CMR was analyzed using Horos (Annapolis, Maryland, USA). LV endocardium and epicardium on end-diastolic and end-systolic sections were traced manually, and thus LV end-diastolic volume (LVEDV) and LV end-systolic volume (LVESV) were calculated [27]. LVEF was determined according to the formula; LVEF = (LVEDV- LVESV)/ LVEDV × 100 (%). LV mass (LVM) (g) was determined as 1.05 g/cm^3^ times LV wall volume. LVM, LVEDV and LVESV were divided by the body weight (BW) to obtain indexed values; LVM/BW, LVEDV/BW, and LVESV/BW, respectively.

The inflammatory area was calculated by Matlab analytic (Mathworks, Natick, Massachusetts, USA). Slices of the mid LV were used to compare image sequences. LV was segmented by tracing epicardial and endocardial contours manually. Mean +/− SD of the signal intensity (SI) was calculated in the remote region of interest (ROI) placed on the skeletal muscle of the chest. All pixels within LV with SI > 2SD higher than the remote ROI was considered positive signal on EGE and LGE images. Negative enhancement with ferumoxytol was evaluated on GRE and myocardium that displayed < 20% of SI of the remote ROI was considered abnormal signal void. This cut-off was selected based on the previous report and the visual correspondence [28]. The inflammatory area was shown as the percentage of the total LV area. T2* was calculated by applying GRE images taken at different TEs to the T2* signal intensity decay formula: I_t_ = I_t = 0_ × e^-TE/T2*^and T2* map was created. Then, T2* and R2* (=1/T2*) of LV were evaluated by tracing LV contours. Finally, the change in R2* of LV, which represents the distribution of ferumoxytol, was determined: delta R2* = R2* (post-contrast)-R2* (pre-contrast).

Contrast to noise ratio (CNR) of EGE, LGE and FE-CMR were calculated to compare the contrast between the lesion and the non-lesion. This was calculated according to the formula:

CNR = (S _(lesion)_ – S _(non-lesion)_) / σ.

S _(lesion)_ = mean SI of the lesion,

S _(non-lesion_) = mean SI of the non-lesion,

σ_(lesion)_ = standard deviation of the lesion.

σ_(non-lesion)_ = standard deviation of the non-lesion.

σ = (1/2) √ (σ^2^_(lesion)_ + σ^2^_(non-lesion)_).

CMR analysis was performed by two researchers and one of them was blinded to the inflammatory phases.

#### Histology

**Fig. 2:**
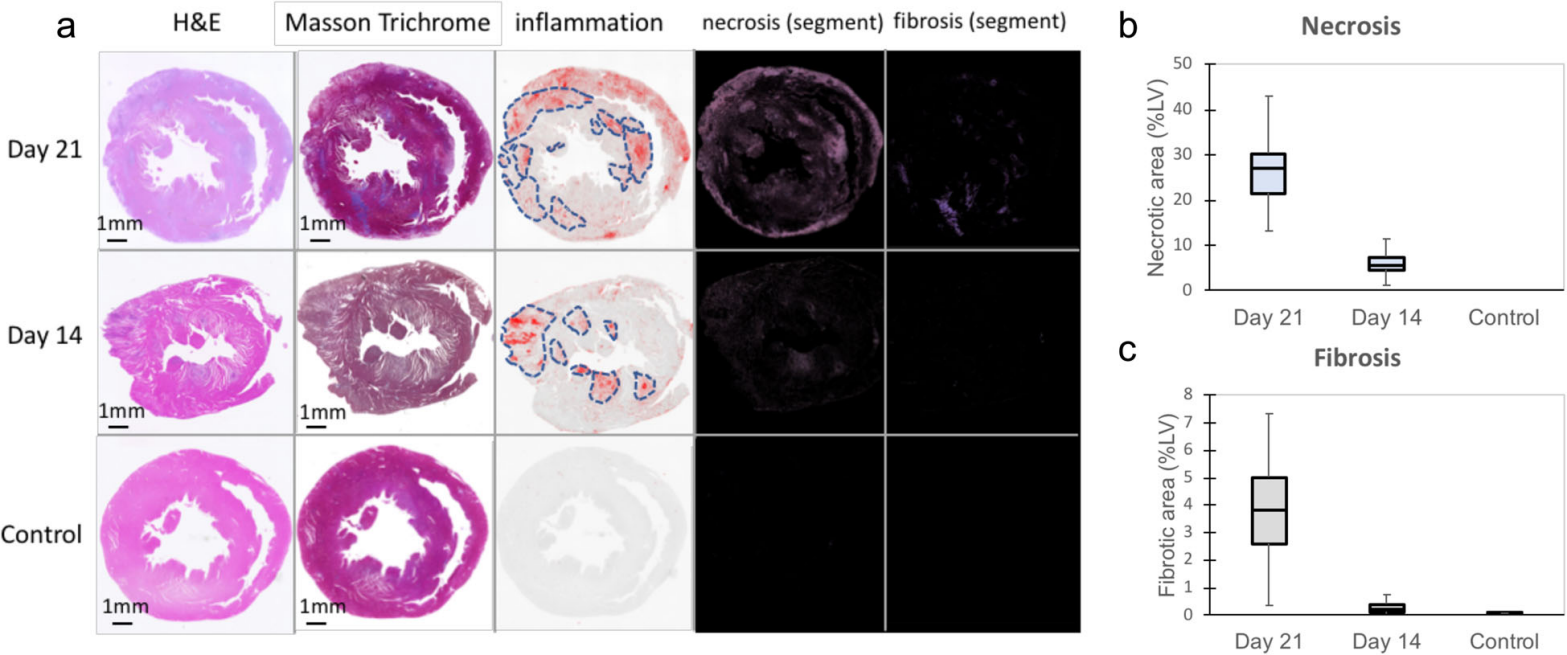
Histological assessment of myocarditis Representative pictures of H&E staining (left column) and Masson Trichrome staining (the second column from the left) from Day 21 (upper row), Day 14 (middle row), and Control (lower row) groups are shown in the panels (**a**). The middle panels (**a**) show segmentations of inflammatory lesions in LV on ImageJ software. The nuclei are highlighted in red in those pictures. The right panels (**a**) show the segmented necrotic area (the second column from the right) and fibrotic area (right column). The necrotic area (**b**) and fibrotic area (**c**) of Day 21, Day 14, and Control hearts were quantified and displayed in the graphs

After the CMR studies, the rats were euthanized by cutting the aorta under anesthesia (5% isoflurane) at day 14 or day 21, respectively for each group and the ex-vivo histological evaluation was performed. The removed hearts were cut into halves. One was fixed in 10% formalin and embedded in paraffin, and the other was embedded in OCT compounds (Sakura Finetek) and snap-frozen. Paraffin-embedded tissues were used for staining with Hematoxylin-Eosin (H&E), Masson’s Trichrome, and Prussian blue to evaluate myocardial inflammatory infiltrates/necrosis, myocardial fibrosis, and iron particle accumulation, respectively. The sections were examined and the pictures were taken using BZ-X700 microscope and its internal software (Keyence Corporation of America, Itasca, Illinois, USA). Areas with inflammatory cellular infiltrates were traced on H&E staining and the ratio to the LV was analyzed using ImageJ. Nuclei were highlighted by the threshold to detect nuclei of myocardium clearly on color-split images. The area with clusters of infiltrating cells were enclosed by polygons (Fig. 2). Thus, the total inflammatory areas were calculated. Fibrosis and necrosis were quantified on Masson Trichrome staining using the software calculated on Matlab. The RGB images were converted to LAB color space and the specific colors of fibrosis or necrotic tissue were selected by placing ROI in representative fibrotic or necrotic regions, respectively. Then, for each pixel, the difference between the pixel’s color and the average LAB color of selected region was calculated. The threshold was set to minimize the overlapping of the colors of segmented regions. Thus, fibrotic and necrotic regions were segmented and calculated (Fig. 2). Ferumoxytol particles distributed in the non-necrotic or necrotic inflammatory regions were counted under high-power field (× 400) of Prussian blue staining slides. The numbers of clearly identifiable intracellular ferumoxytol particles contained in 3 different locations of the non-necrotic or necrotic myocardial inflammation per each rat were summed respectively and analyzed. Frozen tissues were sectioned at the thickness of 7um, fixed with 4% paraformaldehyde, blocked with 3% hydrogen peroxide, and incubated with anti-CD68 (ED1) antibody (mouse monoclonal, abcam, Cambridge, Massachusetts, USA) at 4 °C for overnight. Antibody-HRP conjugate was detected with a Histofine Simplestain system (Nichirei Corporation, Tokyo, Japan) and visualized with DAB substrates. Sections were counterstained with hematoxylin. The number of infiltrating CD68 positive cells were counted on the pictures taken under high magnification (× 100). Ten slices to cover the entire heart were used for each rat (*n* = 5 each for day 21 and day 14 group (50 slices per group) and *n* = 3 for control (30 slices)). The average numbers of infiltrating macrophages per slice were determined. To detect colocalization of macrophage, sections were stained with Prussian blue following immunohistochemistry for CD68 and nuclei were stained with methyl green. Slices obtained at both 6 and 24 h post-ferumoxytol injection were used (*n* = 3 in each group). Histological analysis was performed by two researchers and one of them was blinded to the inflammatory phases.

#### The Miles assay

The enhanced vascular permeability and retention (EPR) effect in the rats at day 14 (n = 3) was evaluated by the Miles assay [29]. In this assay, pathological increase of vascular permeability can be assessed by extravasation of Evans Blue bound to albumin, which is impermeable to the endothelium under normal physiological condition. Evans Blue dissolved in saline (30 mg/kg) was injected through the tail vein and 30 min later, the rats were perfused systemically with saline to wash out Evans Blue dye from the blood. The hearts were sliced and the pictures were taken to assess extravasation of Evans Blue visually.

#### Statistical analysis

All data were expressed as mean ± SD. XLSTAT software (Addinsoft, Paris, France) was used for statistical analysis. The normal distribution of the data was confirmed using Shapiro-Wilk test. Means between two groups were compared using a two tailed t-test for the data with normal distribution. Non-parametric analysis (Mann-Whitney test, two-tailed) were used for the data without normal distribution. Differences with values of *p* < 0.05 were considered significant. Pearson’s correlation coefficient (*r*) was determined to test linear correlation between two sample sets.

## Results

### Development of autoimmune myocarditis

Myocarditis was confirmed histologically in all rats at both days 14 and 21 following the immunization, with inflammatory cellular infiltration quantified as 26.6 ± 8.7% of LV at day 14 and 44.5 ± 17.5% of LV at day 21 (day 14 vs day 21, *p* = 0.03) (Fig. 2). Visually, myocardial necrosis was subtle with no fibrotic replacement found at day 14. However, inflammatory regions at day 21 showed multiple myocardial necrotic foci. The size of necrotic region was calculated as 26.3 ± 9.3% of LV at day 21 and 6.4 ± 3.5% of LV at day 14 (*p* < 0.001 vs day 21). Fibrosis was quantified as 3.7 ± 2.5% of LV at day 21 (*p* = 0.003 vs day 14, *p* = 0.004 vs control), 0.29 ± 0.25% of LV at day 14 (*p* = 0.04 vs control), and 0.056 ± 0.028% of LV in the control. The degree of necrosis and fibrosis in myocarditis at days 14 and 21 had a positive correlation (*r* = 0.85, *p* < 0.001). The LV functional analysis is shown in Table 1. At day 14, LVM/BW was mildly increased (2.22 ± 0.54 mg/g vs the control; 1.46 ± 0.27 mg/g, *p* < 0.01) but LV function was preserved (LVEF; 64.6 ± 7.7%, vs control 66.2 ± 6.1%). Conversely, at the peak stage of day 21, LVM/ BW was more substantially increased in the myocarditis group (LVM/BW 3.01 ± 0.99 mg/g, *p* < 0.01 vs control) and LV function was significantly decreased (LVEF; 47.4 ± 16.4%, *p* < 0.01 vs control).

**Table 1:** LV functional analysis

	Control	Day 14	Day 21
LVEF (%)	66 (6)	65 (8)	47 (16) †
LVM (mg)	345 (33)	348 (72)	545 (156) *
LVM/BW (mg/g)	1.46 (0.27)	2.22 (0.54) †	3.01 (0.99) †
LVEDV (μl)	361 (34)	245 (37) †	309 (22) †
LVEDV/BW (μl/g)	1.52 (0.24)	1.56 (0.22)	1.69 (0.15)
LVESV (μl)	121 (16)	86 (17) †	163 (55)
LVESV/BW (μl/g)	0.50 (0.02)	0.54 (0.13)	0.90 (0.33) †
BW (g)	239 (25)	158 (14) †	183 (15) †

LV function was analyzed on cine CMR. Mean (SD) of LVEF, LVM, LVM/BW, LVEDV, LVEDV/BW, LVESV, LVESV/BW, and BW of the control, early phase (day 14), and peak phase (day 21) groups are shown. [BW = body weight. Statistical analysis; * *p* = 0.01, †*p* < 0.01 compared to the control.] LVEDV, left ventricular end-diastolic volume; LVEF, left ventricular ejection fraction; LVESV, left ventricular end-systolic volume; LVM, left ventricular mass

### CMR detection of myocardial inflammation

**Fig. 3:**
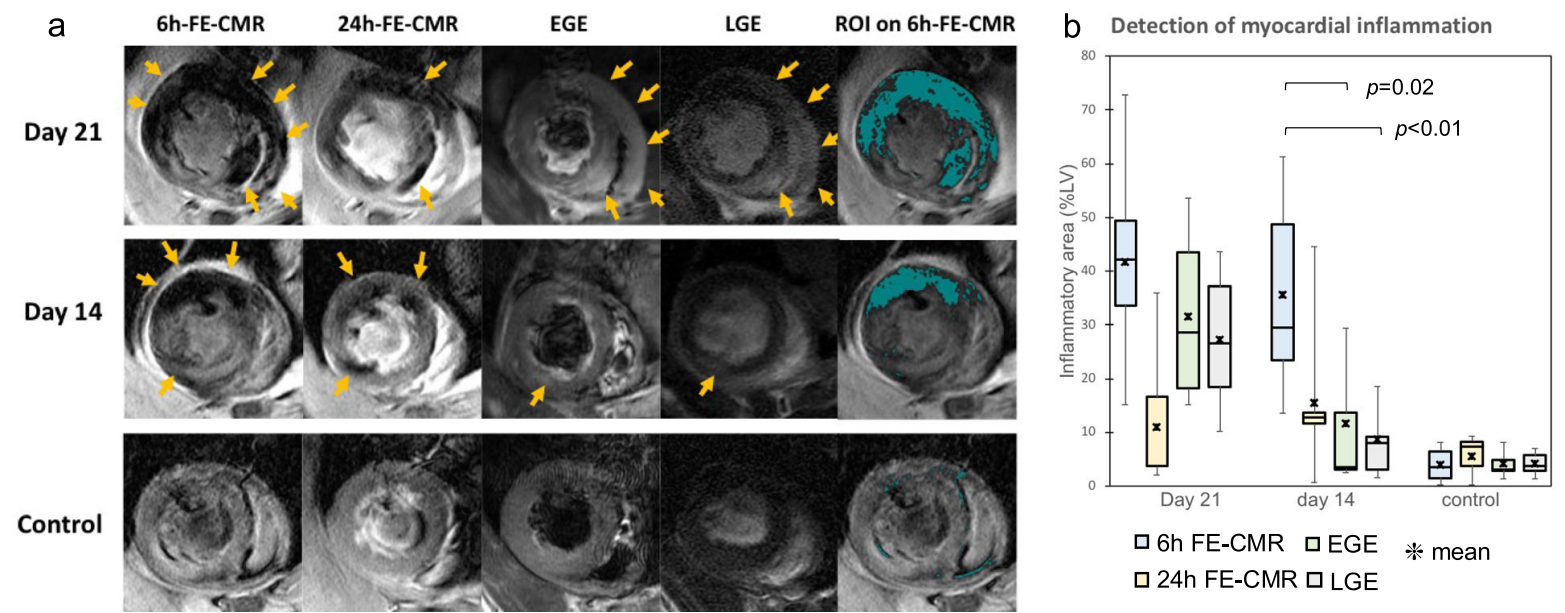
Detection of myocarditis by FE-CMR, early gadolinium enhancement (EGE), and late gadolinium enhancement (LGE) Representative images of FE-CMR performed at 6 h (6 h-FE-CMR) and 24 h (24 h-FE-CMR) after ferumoxytol administration, EGE, and LGE, are shown (**a**) (upper row: day 21, middle row: day14, lower row: control). Enhanced areas suggesting inflammation are pointed by arrows. The right panels show inflammatory lesions (both left ventricle (LV) and right ventricle (RV)) segmented on 6 h-FE-CMR in light blue. The box and whisker graph (**b**) shows the regions detected as myocardial inflammation by 6 h-FE-CMR, 24 h-FE-CMR, EGE, and LGE. At day 14, FE-CMR detected more extensive myocardial inflammation compared with EGE (*p* = 0.02) and LGE (*p* < 0.01)

Representative CMR images of each group are shown in Fig. 3a. The region of myocardial inflammation on each image modality was calculated (Fig. 3b). The SI of the remote ROIs in the skeletal muscle showed comparable values between the control and the EAM groups. At the early phase of day 14, 6 h-FE-CMR could clearly detect the dephasing signal loss in the inflammatory lesions with an average inflammatory area of 33.2 ± 15.0%, which corresponded well with histological inflammatory infiltration. This decreased to 15.4 ± 13.5% on 24 h-FE-CMR (*p* = 0.03 vs 6 h). EGE (11.7 ± 15.5%, *p* = 0.02) and LGE (8.7 ± 8.7%, *p* < 0.01) on day 14 demonstrated significantly decreased detection of myocarditis, which was not significantly different from the areas calculated as false positive in the control: EGE 4.1 ± 2.1%, LGE 4.1 ± 2.1%, and FE-CMR 4.0 ± 3.2% (*p* = 0.001 compared to 6 h-FE-CMR). EGE, LGE and FE-CMR had comparable CNR: 3.94 ± 0.50 (EGE), 4.38 ± 0.35 (LGE), and 3.94 ± 0.37 (FE-CMR) (*p* > 0.05).

At the peak inflammatory phase of day 21, FE-CMR performed at 6 h after the ferumoxytol injection (6 h-FE-CMR) showed extensive and distinct signal loss. Inflammatory area detected on 6 h-FE-CMR was 41.6 ± 18.2%, which showed a larger trend compared to EGE (31.5 ± 15.7%, ns) and LGE (27.2 ± 12.0%, ns). The areas with signal loss detected on 6 h-FE-CMR were significantly decreased, though still detectable, at 24 h (24 h-FE-CMR) (10.9 ± 11.5%, *p* = 0.02 vs. 6 h).

**Fig. 4:**
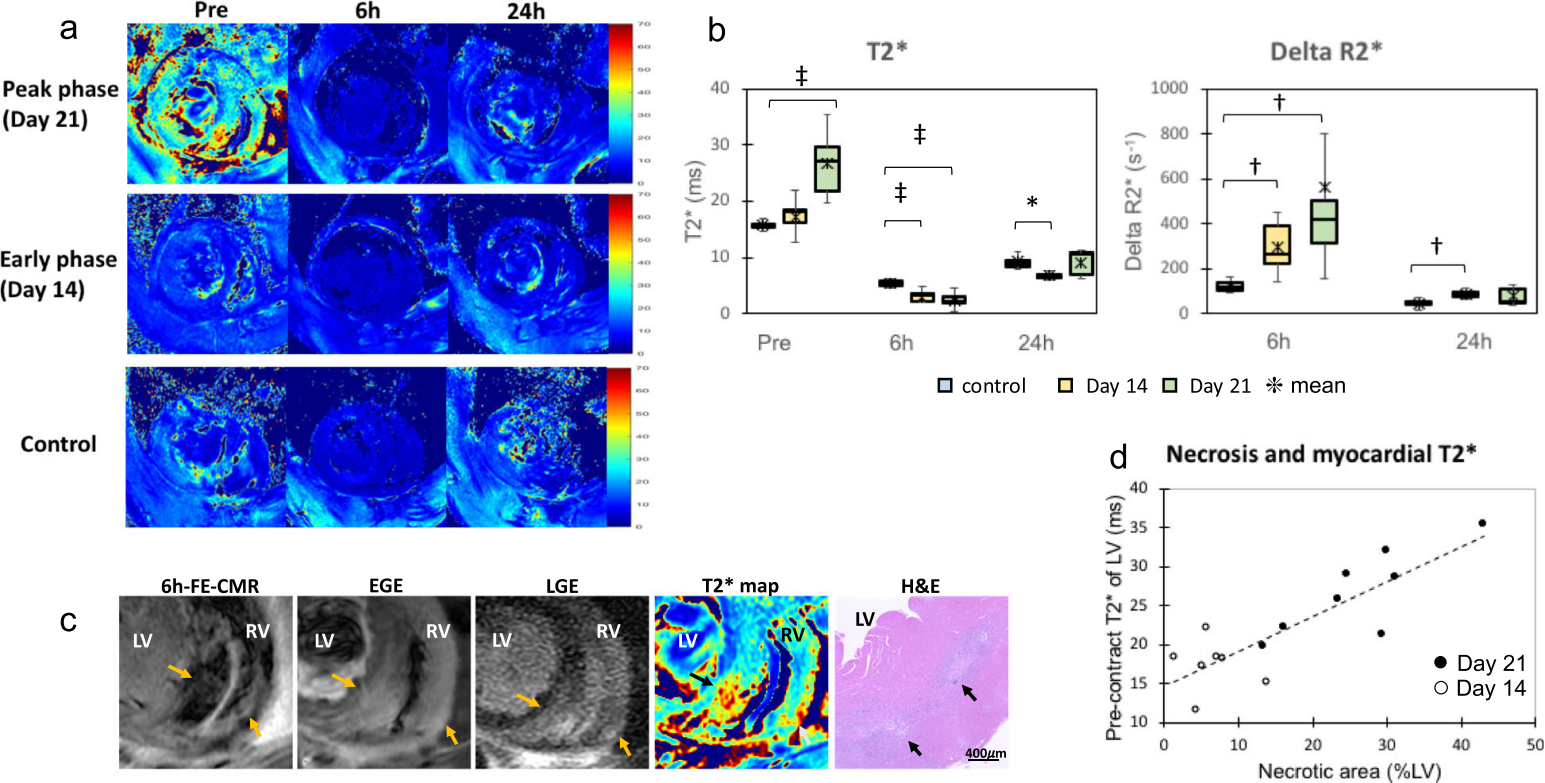
T2* and delta R2* of the myocardium The representative mapping images (**a**) and graphs (**b**) compare the pre- and post-contrast (6 h and 24 h) T2* and delta R2* of LV between the control, day 14, and day 21 groups. The images were obtained from the same rats used in Fig. 3. The day 21 images are magnified to highlight the region with especially high T2* (septum and RV), which showed less ferumoxytol accumulation on 6 h-FE-CMR but high gadolinium accumulation on EGE and LGE (**c**). Histologically, this region corresponded with myocardial necrosis (right panel, arrows). The scatter plot (d) shows a positive linear correlation between the necrotic area size (%LV) and pre-contrast T2* of LV (*r* = 0.86, *p* < 0.001). [statistical analysis; * *p* = 0.01, †*p* < 0.01, ‡*p* < 0.001, compared to Control]

Figure 4 and Table 2 show the pre- and post-contrast T2* and delta R2* of each group. T2* of LV significantly decreased at 6 h-post-contrast both at days 14 and 21 compared to the control. Delta R2* of LV demonstrated the significantly enhanced uptake of ferumoxytol 6 h post-contrast both at day 14 (299 ± 112 s^− 1^, *p* < 0.01) and day 21 (564 ± 562 s^− 1^, *p* < 0.01) compared to the control (125 ± 26 s^− 1^). On the other hand, while significant difference in delta R2* at 24 h-post contrast compared to the control (46 ± 19 s^− 1^) were observed at day 14 (89 ± 18 s^− 1^, *p* < 0.01), significant difference in delta R2* was not confirmed at day 21 (77 ± 39 s^− 1^).

**Table 2:** T2^a^ and delta R2^a^ of myocardium

		Control	Day 14	Day 21
T2* (ms)	Pre	15.9 (0.5)	17.3 (1.2)	26.7 (1.9) ‡
	6 h	5.3 (0.2)	3.0 (0.3) ‡	2.4 (0.2) ‡
	24 h	9.4 (0.7)	6.7 (0.2)*	9.1 (1.0)
Delta R2* (s^− 1^)	6 h	125 (10.5)	299 (42.4) †	564 (198.5) †
	24 h	46 (8)	89 (7) †	77 (17)

T2* and delta R2* of LV obtained pre, and 6 h and 24 h post ferumoxytol injection are shown (control, day 14, and day 21 groups). [statistical analysis; * *p* = 0.01, †*p* < 0.01, ‡*p* < 0.001, compared to Control]

Native T2* of the LV was significantly increased at day 21 compared to control (day 21; 27 ± 5 ms vs control; 16 ± 1 ms, *p* < 0.001), however, no significant change was observed at day 14 (17 ± 3 ms). Histologically, areas with high T2* values corresponded with advanced inflammation accompanying necrosis (Fig. 4c). The quantified necrotic regions had a positive linear correlation with myocardial pre-contrast T2* (*r* = 0.86, *p* < 0.001) (Fig. 4d). The quantified fibrotic regions also had a positive linear correlation with T2* (*r* = 0.82, *p* < 0.001). FE-CMR showed decreased negative contrast effects in areas with increased native T2* corresponding with myocardial necrosis whereas EGE and LGE showed strong positive contrast in those regions (Fig. 4c).

### Iron distribution in the necrotic/ non-necrotic regions

**Fig. 5:**
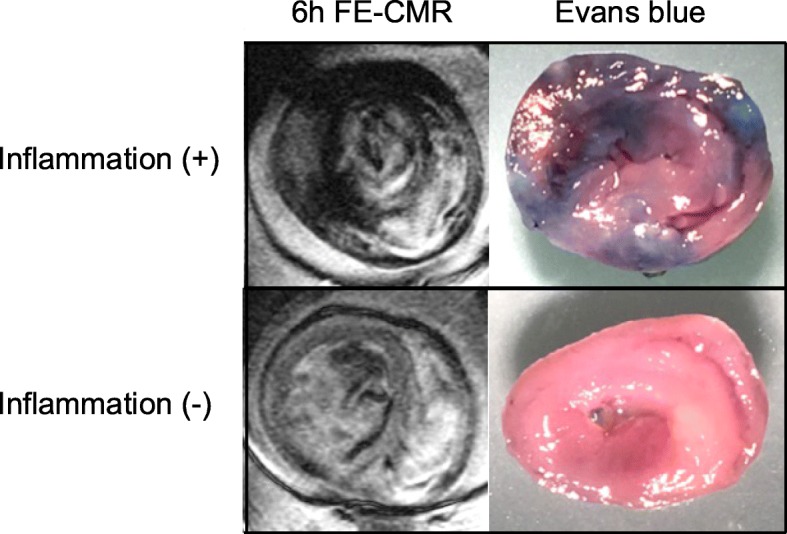
Comparison of iron particle distribution between necrotic and non-necrotic inflammation Iron distribution in the myocardial inflammatory regions was compared between necrotic and non-necrotic tissues on Prussian blue staining (**a**). Necrotic area is highlighted by the dotted line. Prussian blue and H&E staining of the necrotic region (**b** & **c**, respectively) and the non-necrotic regions (**d** & **e**, respectively) clearly show the different distribution of ferumoxytol between those regions. The number of iron particles was counted in the high-power field and compared between the non-necrotic and necrotic inflammatory areas (**e**)

Iron staining with Prussian blue also confirmed that ferumoxytol distributed in the regions with inflammatory cellular infiltration. The inflammatory cellular uptake of ferumoxytol particles was observed in the ex-vivo hearts obtained at 24 h after the ferumoxytol injection. However, the necrotic and non-necrotic inflammation revealed distinctively different distribution of ferumoxytol. Inflammatory tissues with myocardial necrosis contained fewer iron particles while non-necrotic inflammatory tissues showed prominent distribution of iron particles (Fig. 5). Quantification of iron particles demonstrated significantly fewer iron particles in the necrotic tissues (7.2 ± 6.5 particles /high power field) compared to non-necrotic inflammatory tissues (98.3 ± 39.6 particles /high power field, *p* < 0.001) (Fig. 5).

### Iron uptake by macrophages

**Fig. 6:**
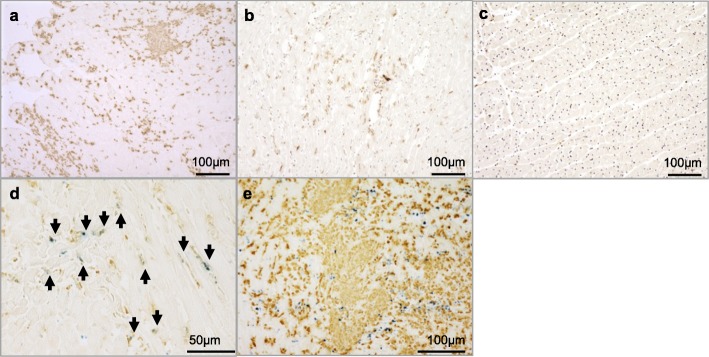
Immunohistochemistry for macrophages Macrophage were detected as CD68 positive cells and visualized with DAB substrate. Macrophage infiltration in the myocardium was confirmed both at Day 21 (**a**) and Day 14 (**b**) but not in Control (**c**). Dual staining of iron particles (Prussian blue) and macrophages (DAB) shows their different distributions between 24 h (**d**) and 6 h (**e**) post-ferumoxytol injection

Macrophage infiltration was confirmed in both Day 14 and 21 EAM hearts (Fig. 6). Day 21 heart had more extensive infiltration of CD68 positive macrophages compared to Day 14 heart (767 ± 201 counts/field vs 282 ± 120 counts/field, *p* < 0.0001). CD68 positive macrophages were scarcely found in the normal control heart (2.8 ± 2.7counts/field). Prussian blue staining and immunohistochemistry showed co-localization of ferumoxytol in some of the CD68 positive macrophages at 24 h post-ferumoxytol injection (Fig. 6). In contrast, Prussian blue staining and immunohistochemistry at 6 h post-ferumoxytol injection confirmed ferumoxytol distribution in the extracellular space adjacent to the infiltrating macrophages (Fig. 6).

### Enhanced vascular permeability and FE-CMR

**Fig. 7:**
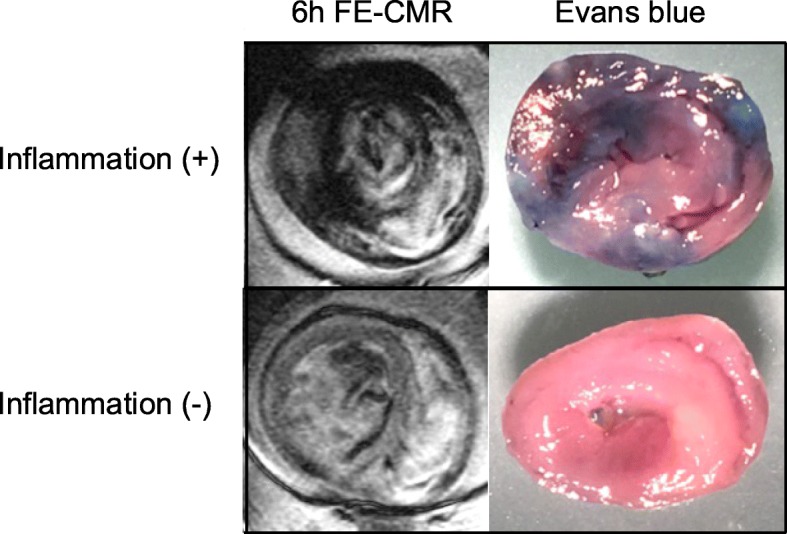
Enhanced vascular permeability and FE-CMR. Inflammatory areas on 6 h-FE-CMR (left panel) and areas with Evans blue distribution (right), which represents enhanced vascular permeability, were compared in the hearts with myocardial inflammation (upper row) and without inflammation (lower row)

The dependence of distribution of ferumoxytol on the enhanced vascular permeability was investigated by visualizing extravasation of Evans Blue in the tissue (Fig. 7). The areas of signal void on 6 h-FE-CMR corresponded with the areas with the myocardium stained blue with Evans Blue’s extravagation in the inflammatory tissue.

## Discussion

This study showed 6 h-FE-CMR has an excellent and superior capability to detect early active myocarditis. Most remarkably, FE-CMR demonstrated a higher sensitivity to the early stage of acute myocarditis compared to EGE and LGE. Conversely, FE-CMR was less sensitive to the necrotic regions in the advanced phase of inflammation while EGE, LGE, and T2* showed high sensitivity to myocardial necrosis. FE-CMR is a promising diagnostic method to discriminate active myocardial inflammation at an early, potentially intervenable stage, which is superior to the detection/treatment window afforded by gadolinium-enhanced CMR.

This study also demonstrated that FE-CMR and T2* mapping at 6 h had a higher sensitivity to myocarditis compared to FE-CMR and T2* mapping at 24 h. A recent clinical study also did not prove the superiority of 24 h-FE-CMR over LGE in the diagnosis of acute myocarditis [30]. Imaging at the earlier time point following ferumoxytol injection could enhance the detection of inflammation. Because of the property of being taken up by phagocytes, images obtained at 24 h or later post injection of iron oxide particles have been widely used to provide functional information by detecting active inflammatory cells, dominated by macrophages, in inflammation-associated diseases including myocardial infarction, acute cardiac transplant rejection, arterial plaques, and tumors [19, 31, 32]. They have also shown the potential of a better sensitivity to smaller and less severe lesions compared to gadolinium-enhanced CMR [25, 32–34]. On the other hand, Yilmaz et al. detected signal loss in the infarct regions of acute myocardial infarction patients as early as 6 h post- ferumoxytol injection, which was explained by the phagocytotic activity of macrophages [21]. In addition, hyperemia and vascular leakage evoked by active inflammation could enhance the distribution of ferumoxytol in the inflammatory sites and promote the subsequent phagocytosis [16]. Ferumoxytol, originally developed as an intravascular contrast agent, has a relatively long plasma half-life time and persists in the blood for a long time. The dextran coating functions to slow the phagocytosis and release the iron from the core [35]. It has been used for contrast CMR angiography, cerebral blood volume mapping, and myocardial perfusion imaging [36–38]. Ferumoxytol is more suitable for enhancing hyperemia and vascular permeability than extravascular gadolinium-based contrast agents [39]. The inflammatory signals on 6 h-FE-CMR observed in this study could have been exaggerated by dynamic enhancement of hyperemia and vascular leakage by ferumoxytol. The correspondence between inflammatory detection on 6 h-FE-CMR and Evans Blue extravasation in our study supports this. High sensitivity of ferumoxytol to myocardial blood flow could also explain its less sensitivity to the tissue necrosis, which has poor blood supply as observed in our study.

Native T1, T2 and ECV mapping have been utilized for diagnosing myocarditis recently. A meta-analysis suggested higher sensitivity of native T1 compared to the Lake Louise Criteria, the current diagnostic criteria [40]. However, otherwise native T1, T2 and extracellular volume fraction (ECV) did not show significant improvement in sensitivity, specificity, or diagnostic odds ratio compared to Lake Louise Criteria. One of the drawbacks of native T1 is it reflects not only acute inflammatory change but also various chronic injuries including fibrosis or infiltrations. Mapping methods also need to be standardized to establish its diagnostic usefulness.

This study also showed correlation of increased pre-contrast T2* with advanced myocarditis. The area with high T2 corresponded with severe inflammation accompanying myocardial necrosis. In addition, the extent of myocardial necrosis had a positive correlation with myocardial T2*. Although, the extent of fibrosis also showed a similar positive correlation with T2*, this was likely to be confounded by the linear correlation between the severity of necrosis and fibrosis at this phase of myocarditis. It is more reasonable that increased T2* reflects necrosis rather than fibrosis, considering that necrosis is dominant at this phase and fibrosis, containing less water content, would be expected to decrease T2* [41]. T2* reflects both T2 and magnetic field heterogeneity, which is affected by the tissue microstructure [42]. Histologically, T2* is known to correlate with tissue water content and collagen fiber network, and the evaluation of T2* has been especially investigated in the diagnosis of cartilage degeneration [42]. The usability of T2* mapping in myocarditis remains unknown. A recent clinical study showed elevated T2* in the hypertrophic myocardium in the hypertrophic cardiomyopathy patients [43]. The increased T2* might result from the T2 change caused by myocardial edema. Because T2* decreases in the fibrotic tissue, T2* mapping could help to stage myocarditis by differentiating acute inflammation from post-myocarditis fibrotic replacement.

Although ferumoxytol has been already translated into clinical application, there have been no established protocols for the dose of ferumoxytol and the timing of imaging to detect inflammatory lesions. The dose of ferumoxytol used in this study (16.8 mgF/kg) was decided based on past animal studies and is higher than the clinical dose applied for perfusion or macrophage imaging (4–11 mgFe/kg). Clinical studies have shown higher doses of ferumoxytol improve image quality [44]. The dose and timing should be optimized in future studies.

### Limitation

This study used a limited number of rats. Although myocarditis was induced in all rats, severity of infiltration and necrosis had variability among individuals. High heart rates of rats could potentially have led to underestimation of the diagnostic ability of gadolinium-enhanced CMR. T2w imaging is routinely used for diagnosis of myocarditis and FE-CMR needs to be compared with T2w imaging. We did not include T2w imaging in our study because of the insufficient image quality resulting from the high heart rate of the rats. However, T2w imaging suffers from inconsistency in diagnosing myocarditis in humans because of the shortcomings including subjective interpretation, low signal to noise ratio, low CNR, slow flow artifact, and variable signal intensity caused by the inversion recovery preparation [45]. Lastly, signal voids produced by ferumoxytol can mask the adjacent anatomic structure and the interpretation could be influenced by hemorrhage or artifacts. Alternative method such as positive contrast visualization of iron-oxide particles should be pursued in the future [46, 47].

## Conclusion

FE-CMR acquired at 6 h enhance detection of early stages of myocarditis before development of necrosis or fibrosis. If this is proven in human clinical studies, it could potentially enable appropriate therapeutic intervention.

## Data Availability

The datasets used and analyzed during the current study are available from the corresponding author on reasonable request.

## Abbreviations

BW: Body weight
CMR: Cardiovascular magnetic resonance
CNR: Contrast to noise ratio
ECG: Electrocardiogram
EGE: Early gadolinium enhancement
EPR: Enhanced vascular permeability and retention
FE: Ferumoxytol-enhanced
GRE: Gradient echo
IR: Inversion recovery
LGE: Late gadolinium enhancement
LV: Left ventricle/left ventricular
LVEDV: Left ventricular end-diastolic volume
LVEF: Left ventricular ejection fraction
LVESV: Left ventricular end-systolic volume
LVM: Left ventricular mass
ROI: Region of interenst
SI: Signal intensity
SPIO: Superparamagnetic iron oxide
T2w: T2-weighted
TE: Echo time
USPIO: Ultra-small SPIO
